# Supplementary material for: Stimulating the Hematopoietic Effect of Simulated Digestive Product of Fucoidan from Sargassum fusiforme on Cyclophosphamide-Induced Hematopoietic Damage in Mice and Its Protective Mechanisms Based on Serum Lipidomics
Source: Mar Drugs. 2022 Mar 9;20(3):201. doi: 10.3390/md20030201 (PMC8950290; doi:10.3390/md20030201)
Supplement: Supplementary file 1 [file marinedrugs-20-00201-s001.zip › marinedrugs-1614684-supplementary.pdf]

Stimulating the hematopoietic effect of simulated digestive product of fucoidan from Sargassum fusiforme on cyclophos-phamide-induced hematopoietic damage in mice and its pro-tective mechanisms based on serum lipidomics

Wei-Ping Ma [1], Shi-Ning Yin [2,3], Jia-Peng Chen [1], Xi-Cheng Geng [1], Ming-Fei Liu [1], Hai-Hua Li [1] and Ming Liu [1,4,*]and Hong-Bing Liu [1,3,4,*]

1 Key Laboratory of Marine Drugs, School of Medicine and Pharmacy, Ocean University of China, Qing-dao 266003, China. maweiping1990@163.com (Wei-Ping Ma); 21180831069@stu.ouc.edu.cn (Jia-Peng Chen); 21200811111@stu.ouc.edu.cn (Xi-Cheng Geng); 1661701601@qq.com (Ming-Fei Liu); shaixuan@ouc.edu.cn (Hai-Hua Li); lmouc@ouc.edu.cn (Ming Liu); liuhongb@ouc.edu.cn (Hong-Bing Liu).

2 Qingdao Institute for Food and Drug Control, Qingdao 266000, China. yinshining@126.com (Shi-Ning Yin).

3 NMPA Key Laboratory for Quality Research and Evaluation of Marine Traditional Chinese Medicine, Qingdao 266000, China.

4 Laboratory for Marine Drugs and Bioproducts, Pilot National Laboratory for Marine Science and Tech-nology, Qingdao 266237, China.

** Correspondence: +86 532 82031823, e-mail: liuhongb@ouc.edu.cn (Hong-Bing Liu); Tel.: +86 532 82031980, e-mail: lmouc@ouc.edu.cn (Ming Liu)*

Table S1. Effects of simulated digestive product of fucoidan from *Sargassum fusiforme* (DSFF) on the body weight gain.

Groups	First day	Third day	Fifth day	Seventh day	Eighth day
Control	23.12 ± 2.33	27.09 ± 1.86	27.90 ± 1.79	28.09 ± 2.02	28.49 ± 2.14
Model	23.00 ± 2.77	23.07 ± 1.37**	24.37 ± 1.92**	23.67 ± 1.32**	24.24 ± 1.86**
rhG-CSF	24.50 ± 2.76	23.73 ± 2.18**	24.95 ± 2.22*	25.26 ± 2.40*	26.39 ± 2.32#
1.8 mg/kg	23.67 ± 2.63	23.57 ± 1.14**	23.13± 2.39**	24.27 ± 1.56**	24.66 ± 1.77**
3.6 mg/kg	24.15 ± 2.91	23.49 ± 1.83**	24.25 ± 1.89**	24.42 ± 2.19**	25.99 ± 3.19
7.2 mg/kg	22.85 ± 2.51	22.92 ± 2.04**	24.03 ± 1.45**	23.69 ± 2.37**	24.05 ± 4.41**

Data are the mean ± standard deviation (n=10). * $p < 0.05$, ** $p < 0.01$, vs. control; # $p < 0.05$, vs. model.
